# Number and ratio of metastatic lymph nodes impacts the prognosis of submandibular gland cancer

**DOI:** 10.1371/journal.pone.0296040

**Published:** 2023-12-29

**Authors:** Qigen Fang, Liyuan Dai, Xu Zhang, Ruihua Luo, Junhui Yuan

**Affiliations:** 1 Department of Head Neck and Thyroid, The Affiliated Cancer Hospital of Zhengzhou University & Henan Cancer Hospital, Zhengzhou, China; 2 Department of Radiology, The Affiliated Cancer Hospital of Zhengzhou University & Henan Cancer Hospital, Zhengzhou, China; Fondazione Policlinico Universitario Agostino Gemelli IRCCS, ITALY

## Abstract

This study aimed to assess the impact of the number and ratio of metastatic lymph nodes (LNs) on prognosis in submandibular gland cancer. To this end, patients were selected from the Surveillance, Epidemiology, and End Results database retrospectively. The effect of the number and ratio of metastatic LNs and the American Joint Committee on Cancer (AJCC) N stage on disease-specific survival (DSS) and overall survival (OS) was analyzed. In addition, prognostic models based on LN evaluation methods were developed to predict the OS and DSS. A total of 914 patients were included. Binary recursive partitioning analysis determined the optimal cut-off number of metastatic LNs (0 vs. 1–2. vs. 3+). The presence of 3+ metastatic LNs carried the greatest impact on prognosis, followed by 1–2 positive LNs occurrences. The ratio of metastatic LNs was an independent factor for DSS and OS. The model had a higher likelihood ratio and C-index than those in the Cox model based on the AJCC N stage. Quantitative LN burden and ratio of metastatic LNs provides better survival stratification than the AJCC N stage.

## Introduction

Submandibular gland cancer (SGC) is relatively uncommon and accounts only for approximately 12% of all major salivary gland malignancies [1]. Lymph node (LN) metastasis is not frequent in SGC but exhibits an essential impact on prognosis. Current neck status is evaluated using the American Joint Committee on Cancer (AJCC) classification, which is formulated based on the head and neck squamous cell carcinoma [2]. Its compliance with salivary gland cancer is questioned owing to the different biologic behaviors between the two kinds of disease [3].

Several authors have reported the superiority of proposed N stages based on the number or ratio of metastatic LNs to the AJCC N stage in predicting survival in salivary gland cancer [4–6]. However, all these studies do not list SGC as an independent variable, and SGC shows distinct histologic and prognosis patterns with other salivary gland cancers [7]. Validation of the significance of the number and ratio of metastatic LNs in SGC is warranted; however, only one study analyzed the association between these variables and prognosis, and it was limited by a small sample size [8].

Therefore, the present study aimed to evaluate the impact of the number and ratio of metastatic LNs on survival in SGC using the large Surveillance, Epidemiology, and End Results (SEER) database.

## Materials and methods

### Patient selection

**Fig 1:**
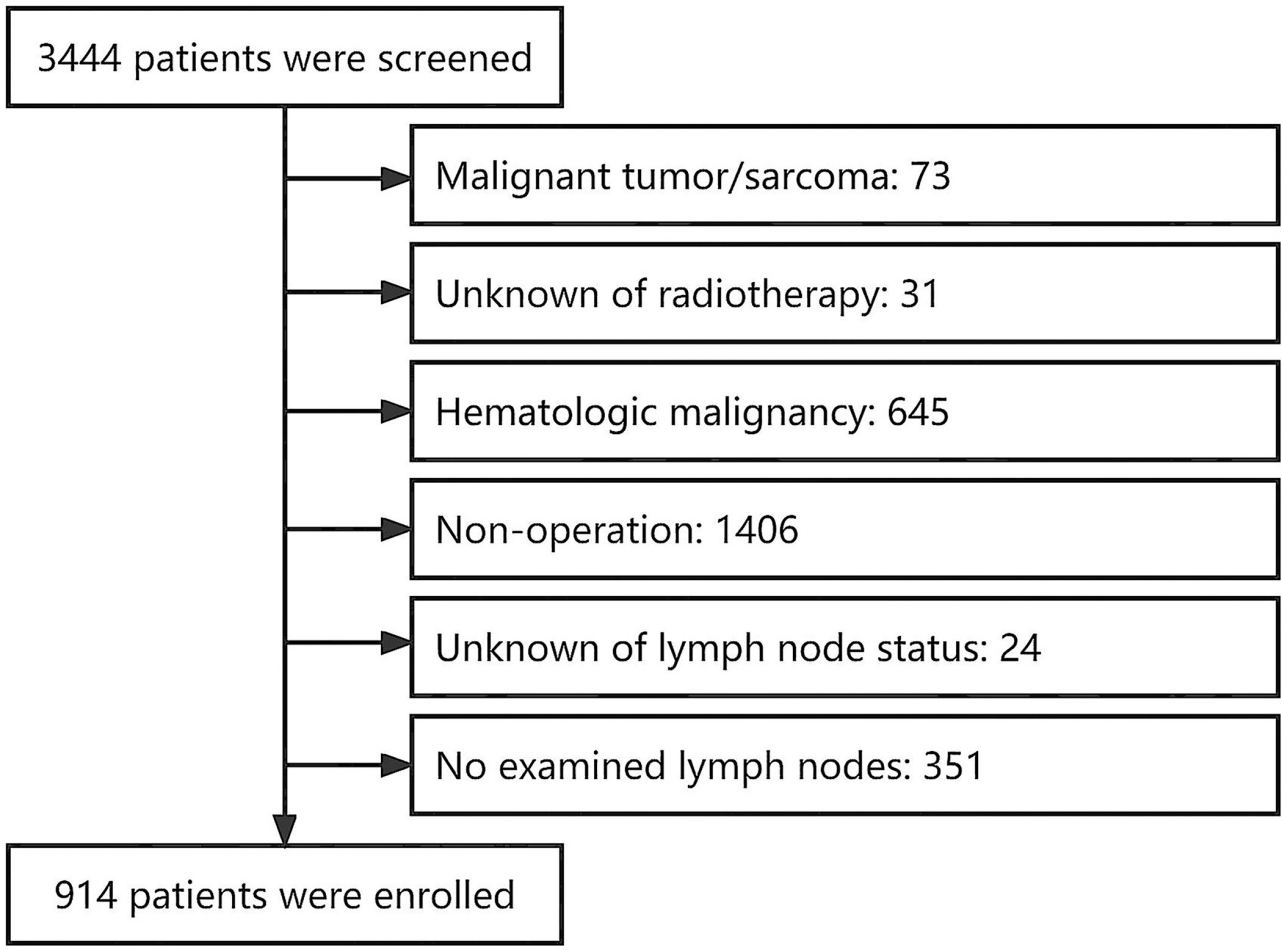
Flowchart of enrolled patients.

All data were obtained from the SEER database [incidence-SEER research data, 17 registries, November 2022 Sub (2000–2020)] on June 6, 2023. This database provides information on cancer statistics in an effort to reduce the cancer burden among the United States population [9]. A total of 3,444 patients with primary submandibular gland malignancy were screened. Of these 3,444 patients, 2,530 were excluded (malignant tumor or sarcoma: n = 73; unknown of radiotherapy: n = 31; hematologic malignancy: n = 645; non-operation: n = 1406; unknown LN status: n = 24; and number of examined LNs smaller than 4: n = 351) (Fig 1). Finally, 914 patients were enrolled. Information regarding patients’ demographics, pathology, treatment, and follow-up was extracted.

### Variable definitions

Histologic grade was classified as low for well-differentiated, intermediate for moderately differentiated, and high for poorly or undifferentiated tissues. Radiotherapy was defined as external beam radiation therapy. Tumor size was determined based on the CS tumor size (2004–2015), extent of disease (EOD) 10-size (1988–2003), and EOD 4-size (1983–1987). The number and ratio of positive LNs were calculated based on the regional nodes examined (1988+) and regional nodes positive (1988+). Pathologic types were analyzed by adenoid cystic carcinoma (ACC) vs. mucoepidermoid carcinoma (MEC) vs. others. The 8^th^ AJCC N stage was formulated according to derived EOD 2018 N (2018+), derived AJCC N 7^th^ edition (2010–2015), derived SEER combined N (2016–2017), and derived AJCC N 6^th^ edition (2004–2015) with consideration of LN size records (2010+), EOD regional nodes (2018+), CS LNs (2004–2015), and CS reg node eval (2004–2015).

Primary outcome variables were overall survival (OS) and disease-specific survival (DSS); OS time was calculated from the date of surgery to that of death or final follow-up, and DSS time was calculated from the treatment date to that of cancer-caused death or final follow-up.

### Statistical analyses

Three models based on different LN evaluation methods were constructed to predict the OS and DSS. In the model 1, the LN status was assessed as 0 vs. 1 vs. 2 vs. 3 vs. 4 vs. 5 vs. 6. vs. 7+ positive LNs, in model 2, the LN status was analyzed according to the result of binary recursive partitioning analysis, in model 3, the LN status was evaluated using the 8^th^ AJCC N stage. In each model, univariate analysis was performed to identify significant factors which were further included in the multivariate analysis to detect independent variables, and the hazard ratios (HRs) for each level of LN status were calculated using Cox proportional hazard models.

Prognostic accuracy between model 2 and model 3 was measured using hazard discrimination and consistency. Hazard discrimination refers to a difference in outcomes between patients in different subgroups, who should have demonstrably different outcomes. It was reflected using Harrell’s C-concordance index; the higher the value, the better the discrimination. Hazard consistency refers to the homogeneity of patients within the same subgroup, who should have similar outcomes. It was reflected by the likelihood ratio, and a good hazard consistency was indicated if the *P*-value was > 0.5.

All statistical analyses were performed using R version 3.4.3 (R Foundation for Statistical Computing, Vienna, Austria). Statistical significance was set at *P* < 0.05.

## Results

### Baseline data

Among the 914 patients, the mean age was 58 ± 19 years; there were 496 (54.3%) men and 418 (45.7%) women. Of the 914 patients, 58.8% were married at initial treatment, and most (58.8%) were white. Pathologic grade was low in 57 (6.2%) patients, intermediate in 153 (16.7%), and high in 329 (36.0%). ACC and MEC had comparable frequency (32.7% vs. 32.9%). Most (94.2%) surgeries were conducted within 1 month after diagnosis. Tumor size was no more than 2 cm in 229 (25.1%) patients and greater than 4 cm in 195 (21.3%). Adjuvant radiotherapy and chemotherapy were performed in 642 (70.2%) and 159 (17.4%) patients, respectively. The mean follow-up time was 62.8 ± 59.0 months. A total of 431 deaths occurred, of which 283 were caused by cancer.

LN metastasis developed in 382 (41.8%) patients. The median ratio of positive to total examined LNs was 0.21 (range: 0–1.0). The number of metastatic LNs was 1 in 103 (11.3%) patients, 2 in 75 (8.2%), 3 in 40 (4.4%), 4 in 19 (2.1%), 5 in 23 (2.5%), 6 in 23 (2.5%), and 7 or more in 99 (10.8%).

### Optimal cut-off number of metastatic LNs

**Fig 2:**
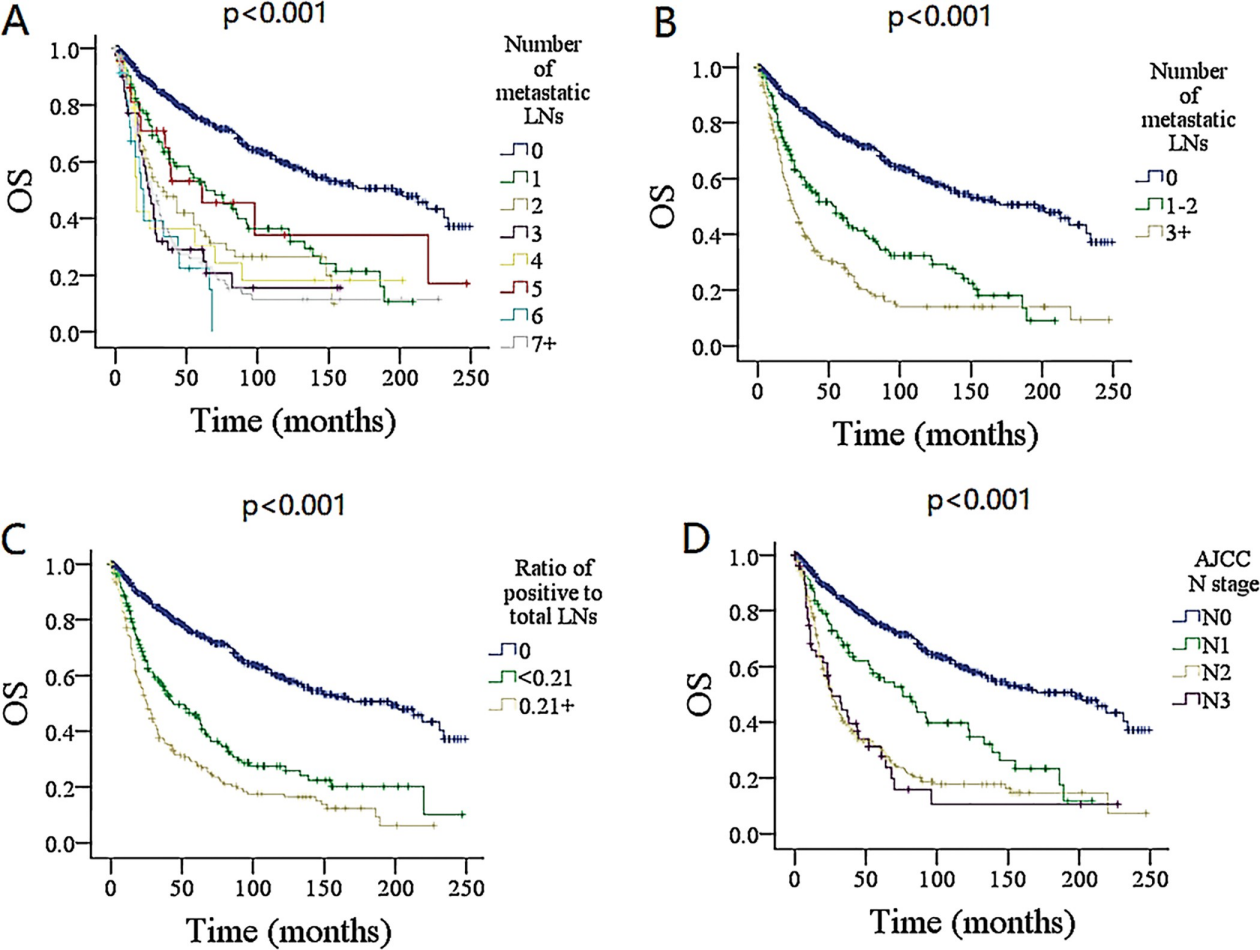
Comparison of overall survival (OS) among patients with different lymph node (LN) features. A for 0 vs. 1 vs. 2 vs. 3 vs. 4 vs. 5 vs. 6 vs. 7+; B for 0 vs. 1–2 vs. 3+; C for ratio of positive to total lymph nodes; D for AJCC N stage. AJCC, American Joint Committee on Cancer.

**Fig 3:**
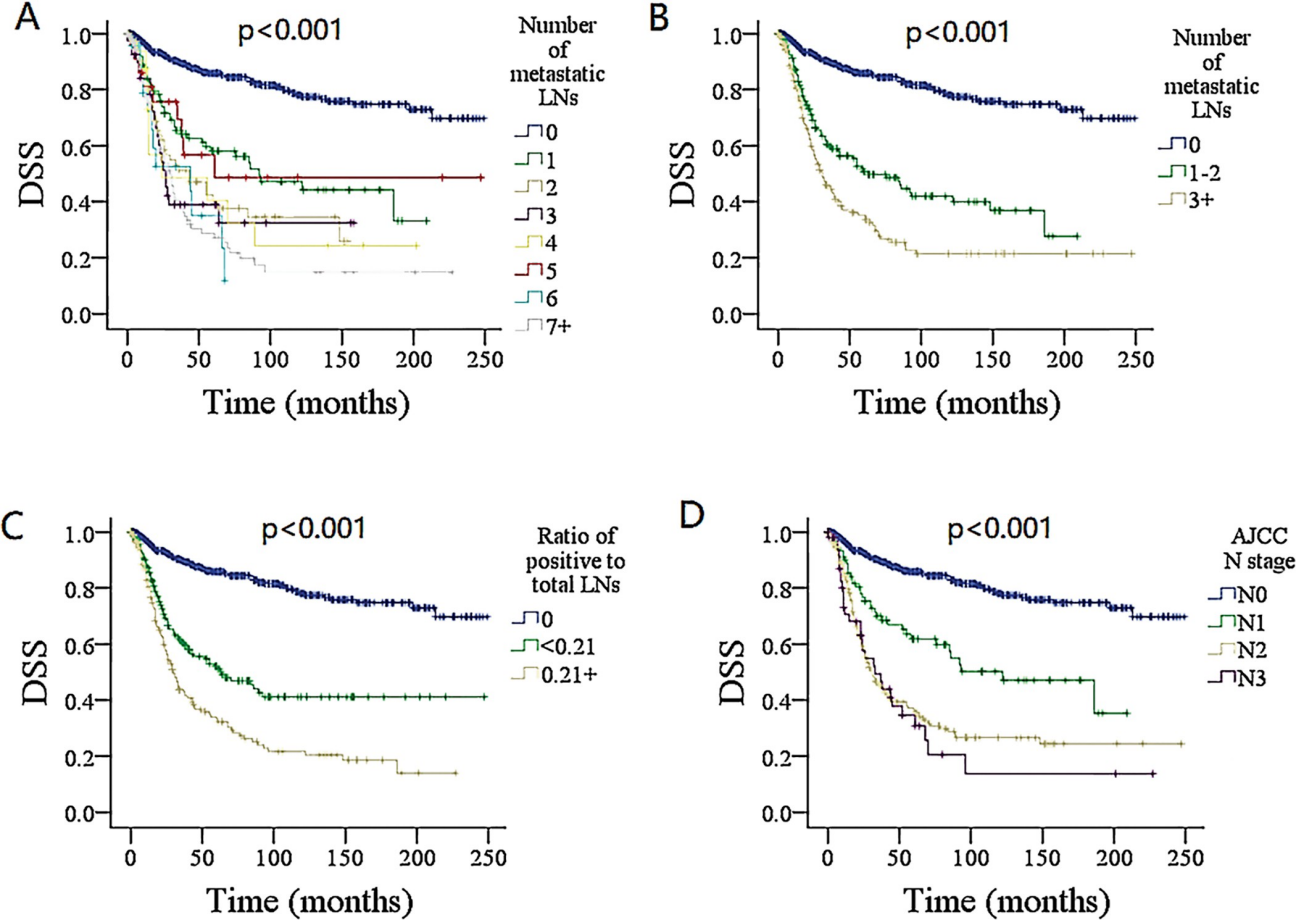
Comparison of disease specific survival (DSS) among patients with different lymph node (LN) features. A for 0 vs. 1 vs. 2 vs .3 vs. 4 vs. 5 vs. 6 vs. 7+; B for 0 vs. 1–2 vs. 3+; C for ratio of positive to total lymph nodes; D for AJCC N stage. AJCC, American Joint Committee on Cancer.

In univariate analysis, variables, including age, sex, race, pathologic grade and type, adjuvant chemotherapy, ratio of positive to total LNs, tumor size, number of metastatic LNs (Figs 2 and 3), and the AJCC N stage were related to DSS and OS (all *P* < 0.05) (Table 1), these significant factors except for the AJCC N stage were evaluated further in the models 1 and 2, and these factors except for the number and ratio of positive LNs were further assessed in model 3.

**Table 1:** Univariate analysis of prognostic factors for disease specific survival (DSS) and overall survival (OS).

Variable	DSS	OS
Age (~39 vs 40–59 vs 60+)	<0.001	<0.001
Sex (Male vs female)	<0.001	<0.001
Marital status (Married vs others)	0.479	0.156
Race (White vs others)	0.021	0.009
Pathologic grade (Low vs intermediate vs high)	<0.001	<0.001
Pathologic type (ACC vs MEC vs others)	<0.001	<0.001
Adjuvant radiotherapy (Yes vs no)	0.018	0.508
Adjuvant chemotherapy (Yes vs no)	<0.001	<0.001
Time to surgery (~1 month vs 2+ months)	0.256	0.069
Ratio of positive to total lymph nodes (0 vs ~0.21 vs 0.21+)	<0.001	<0.001
Tumor size (~2cm vs 2-4cm vs 4+cm)	<0.001	<0.001
Number of metastatic LNs (0 vs 1 vs 2 vs 3 vs 4 vs 5 vs 6 vs 7+)	<0.001	<0.001
Number of metastatic LNs (0 vs 1–2 vs 3+)	<0.001	<0.001
8^th^ AJCC N stage (N0 vs N1 vs N2 vs N3)	<0.001	<0.001

In the model 1, the number and ratio of metastatic LNs, tumor size, and pathologic grade remained independent in predicting DSS and OS. Compared with non-positive LN, LN metastasis significantly decreased the DSS and OS, and different LN burdens had different impacts on DSS and OS. Although their 95% confidence interval (CI) overlapped to some extent, it seemed that the higher was the number, the greater the HR. Meanwhile, the HR in the group with a ratio ≥ 0.21 was higher than those in the other two groups. (Tables 2 and 3)

**Table 2:** Independent impact of metastatic lymph nodes (LNs), ratio of positive to total LNs and the 8^th^ AJCC N stage on disease specific survival.

	p	HR[95%CI]
**Model 1**		
Number of metastatic LNs		
0		
1	0.012	2.38 [1.21–4.70]
2	0.004	2.60 [1.36–4.97]
3	0.003	3.24 [1.50–6.98]
4	0.001	4.86 [1.88–12.58]
5	0.020	3.84 [1.24–11.92]
6	<0.001	7.78 [3.31–18.28]
7+	<0.001	7.35 [5.28–10.24]
Ratio of positive to total LNs		
0		
<0.21	<0.001	3.94 [2.91–5.34]
≥0.21	<0.001	6.57 [4.95–8.74]
**Model 2**		
0		
1–2	<0.001	4.03 [2.97–5.47]
3+	<0.001	6.37 [4.80–8.47]
Ratio of positive to total LNs		
0		
<0.21	<0.001	3.94 [2.91–5.34]
≥0.21	<0.001	6.57 [4.95–8.74]
**Model 3**		
N0		
N1	<0.001	3.026 [2.05–4.46]
N2	<0.001	5.99 [4.54–7.91]
N3	<0.001	6.85 [4.50–10.42]

**Table 3:** Independent impact of metastatic lymph nodes (LNs), ratio of positive to total LNs and the 8^th^ AJCC N stage on overall survival.

	p	HR[95%CI]
**Model 1**		
Number of metastatic LNs		
0		
1	<0.001	2.33 [1.74–3.12]
2	<0.001	3.23 [2.34–4.48]
3	<0.001	4.51 [3.05–6.66]
4	<0.001	3.67 [2.13–6.34]
5	0.011	2.15 [1.20–3.86]
6	<0.001	6.32 [3.87–10.33]
7+	<0.001	4.41 [3.34–5.81]
Ratio of positive to total LNs		
0		
<0.21	<0.001	2.71 [2.14–3.43]
≥0.21	<0.001	4.08 [3.25–5.11]
**Model 2**		
Number of metastatic LNs		
0		
1–2	<0.001	2.63 [2.07–3.35]
3+	<0.001	4.11 [3.29–5.13]
Ratio of positive to total LNs		
0		
<0.21	<0.001	2.71 [2.14–3.43]
≥0.21	<0.001	4.08 [3.25–5.11]
**Model 3**		
N0		
N1	<0.001	2.13 [1.56–2.90]
N2	<0.001	3.86 [3.11–4.79]
N3	<0.001	4.15 [2.88–5.99]

Based on the binary recursive partitioning analysis, the patients were divided into three categories (0 vs. 1–2 vs. 3+). In model 2, the presence of 3+ metastatic LNs had an HR of 6.37 (95% CI: 4.80–8.47) for DSS and an HR of 4.11 (95% CI: 3.29–5.13) for OS; it carried the greatest impact on prognosis among the three groups. Compared with groups with a ratio < 0.21 and 0, those with a ratio ≥ 0.21 had the greatest HR for both DSS and OS (Tables 2 and 3). Other independent factors included age, tumor size, and pathologic grade (Table 4). The model had a likelihood ratio of 0.504 for DSS, 0.497 for OS, and a C-index of 0.788 for DSS, 0.777 for OS.

**Table 4:** Other independent variables in different models for disease specific survival and overall survival (OS).

Variable	DSS	OS
**Model 1**	HR[95%CI]	HR[95%CI]
Pathologic grade		
Low		
Intermediate	1.98[1.16–2.35]	1.68[1.21–2.05]
High	3.87[1.68–5.29]	2.77[1.34–3.80]
Tumor size		
~2cm		
2-4cm	1.76[1.12–2.12]	1.54[1.10–1.98]
4+cm	2.25[1.36–3.27]	1.98[1.34–2.87]
**Model 2**		
Age		
~39		
40–59	1.06[0.56–1.36]	1.04[0.67–1.40]
60+	1.11[0.82–1.58]	1.10[1.04–1.39]
Pathologic grade		
Low		
Intermediate	1.58[1.13–2.26]	1.53[1.17–2.10]
High	3.16[1.45–4.10]	2.68[1.27–3.53]
Tumor size		
~2cm		
2-4cm	1.54[1.08–2.21]	1.26[1.08–1.74]
4+cm	2.31[1.40–3.18]	1.93[1.25–2.67]
**Model 3**		
Age		
~39		
40–59	1.04[0.62–1.43]	1.07[0.52–1.39]
60+	1.20[0.76–1.64]	1.11[1.02–1.42]
Pathologic grade		
Low		
Intermediate	1.67[1.23–2.33]	1.74[1.19–2.47]
High	3.42[1.27–4.98]	2.42[1.28–3.76]
Tumor size		
~2cm		
2-4cm	1.54[1.15–2.21]	1.37[1.09–1.78]
4+cm	2.00[1.27–3.45]	1.88[1.21–2.92]

### Comparison between the number and ratio of metastatic LNs and the AJCC N stage

In the model 3, the AJCC N stage posed an independent effect on DSS and OS (Tables 2 and 3). Higher N stage exhibited more survival compromise, but stages of N2 and N3 had similar HRs, and their 95% CI had obvious overlap. Other independent variable consisted of age, tumor size, and pathologic grade (Table 4). The model had a likelihood ratio of 0.417 for DSS, 0.411 for OS, a C-index of 0.690 for DSS, 0.688 for OS, and showed inferior survival stratification to model 2.

### Subgroup analysis

**Fig 4:**
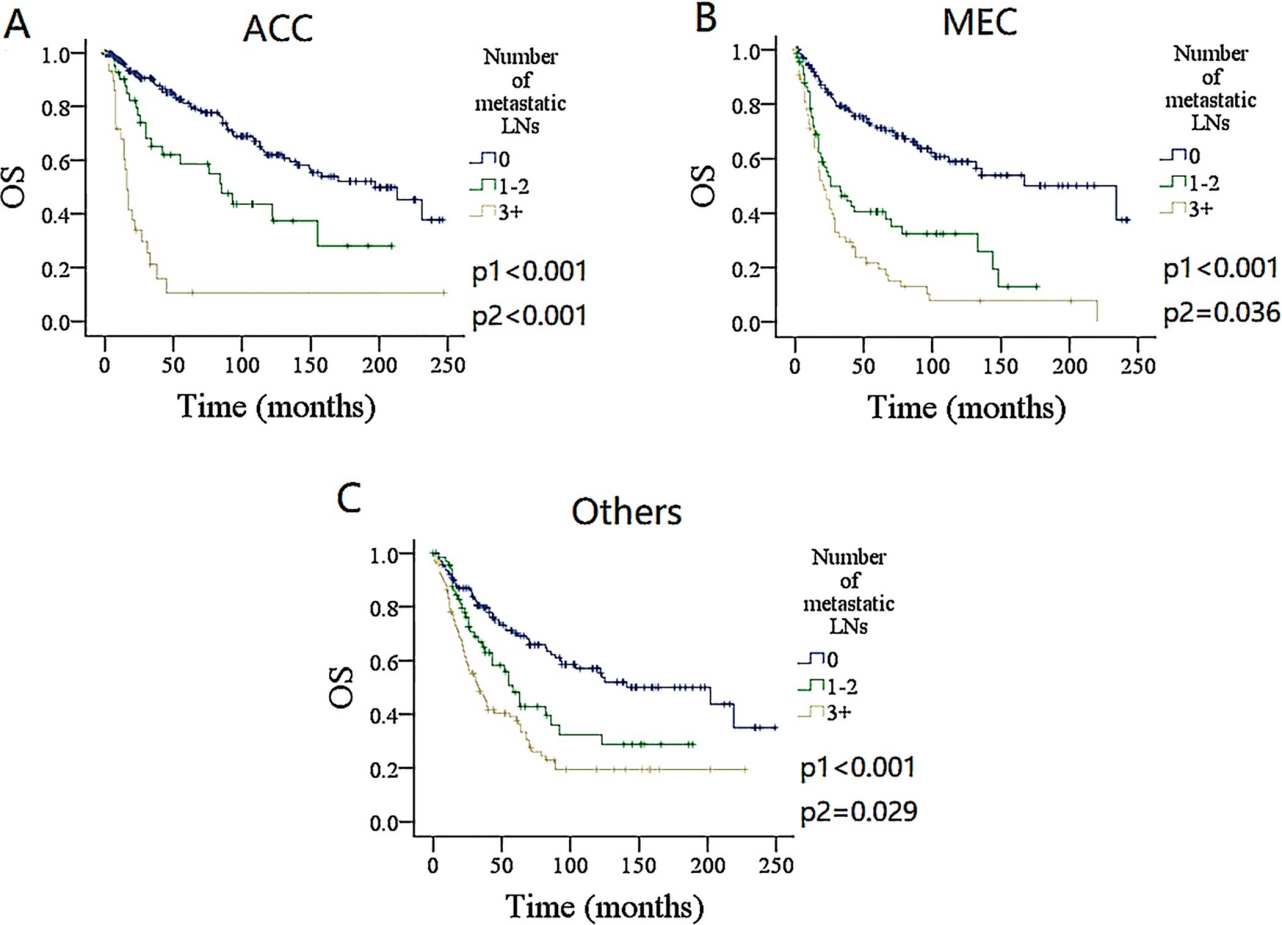
Comparison of overall survival (OS) among patients with different number of metastatic lymph nodes (LNs) stratificated by pathologic types. A for adenoid cystic carcinoma (ACC), B for mucoepidermoid carcinoma (MEC), C for other types.

**Fig 5:**
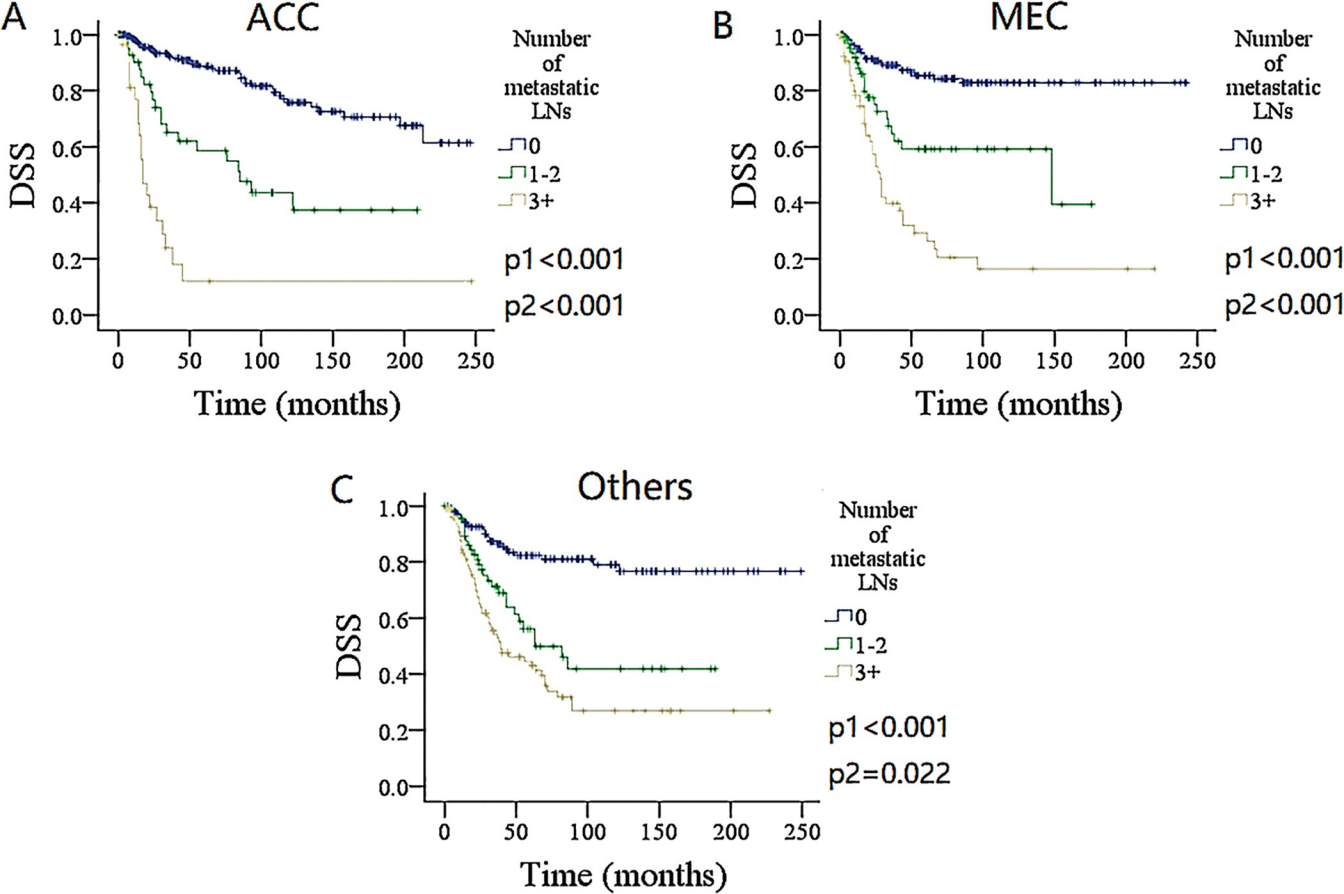
Comparison of disease specific survival (DSS) among patients with different number of metastatic lymph nodes (LNs) stratificated by pathologic types. A for adenoid cystic carcinoma (ACC), B for mucoepidermoid carcinoma (MEC), C for other types.

**Fig 6:**
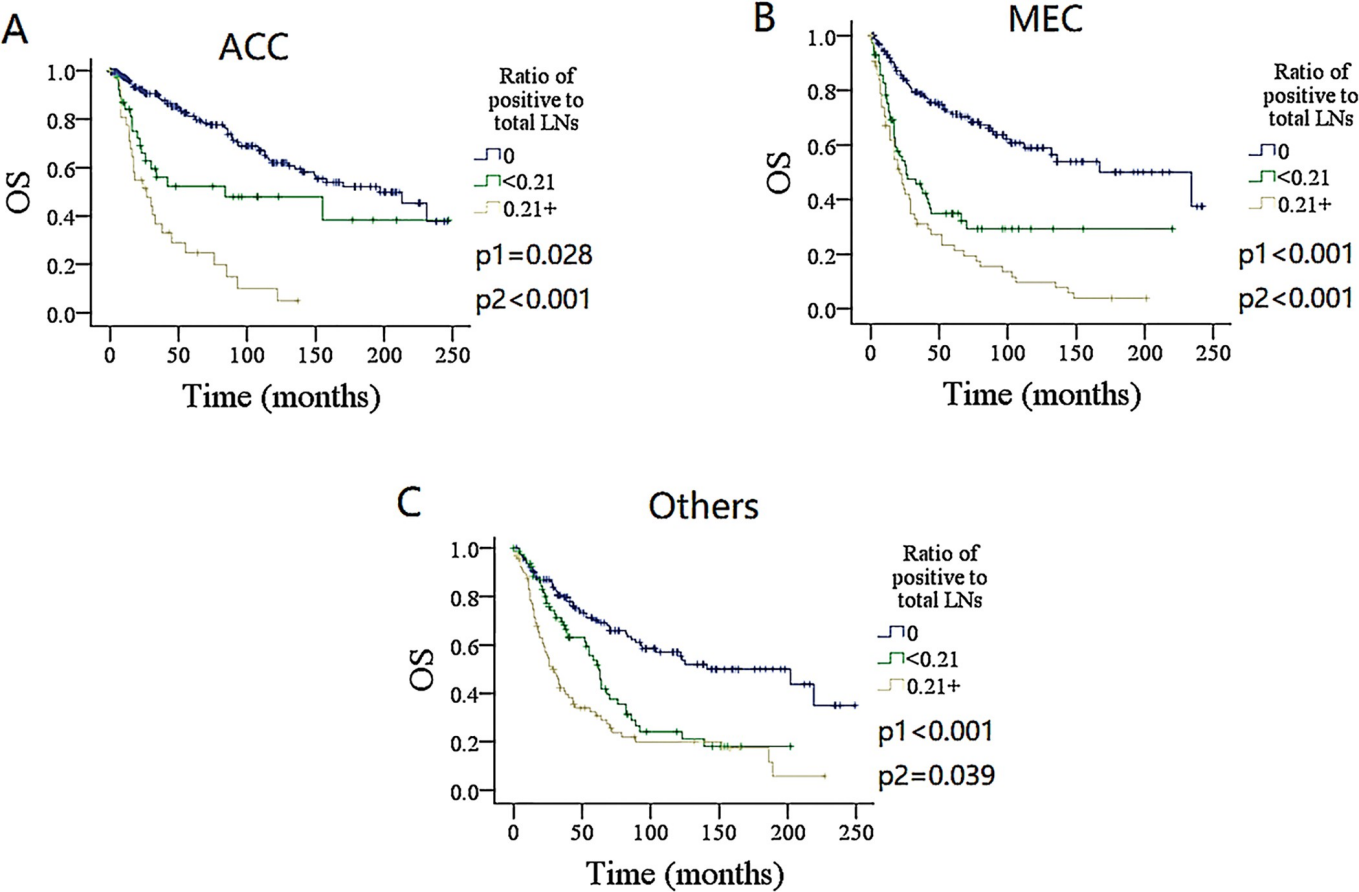
Comparison of overall survival (OS) among patients with different ratio of metastatic lymph nodes (LNs) stratificated by pathologic types. A for adenoid cystic carcinoma (ACC), B for mucoepidermoid carcinoma (MEC), C for other types.

**Fig 7:**
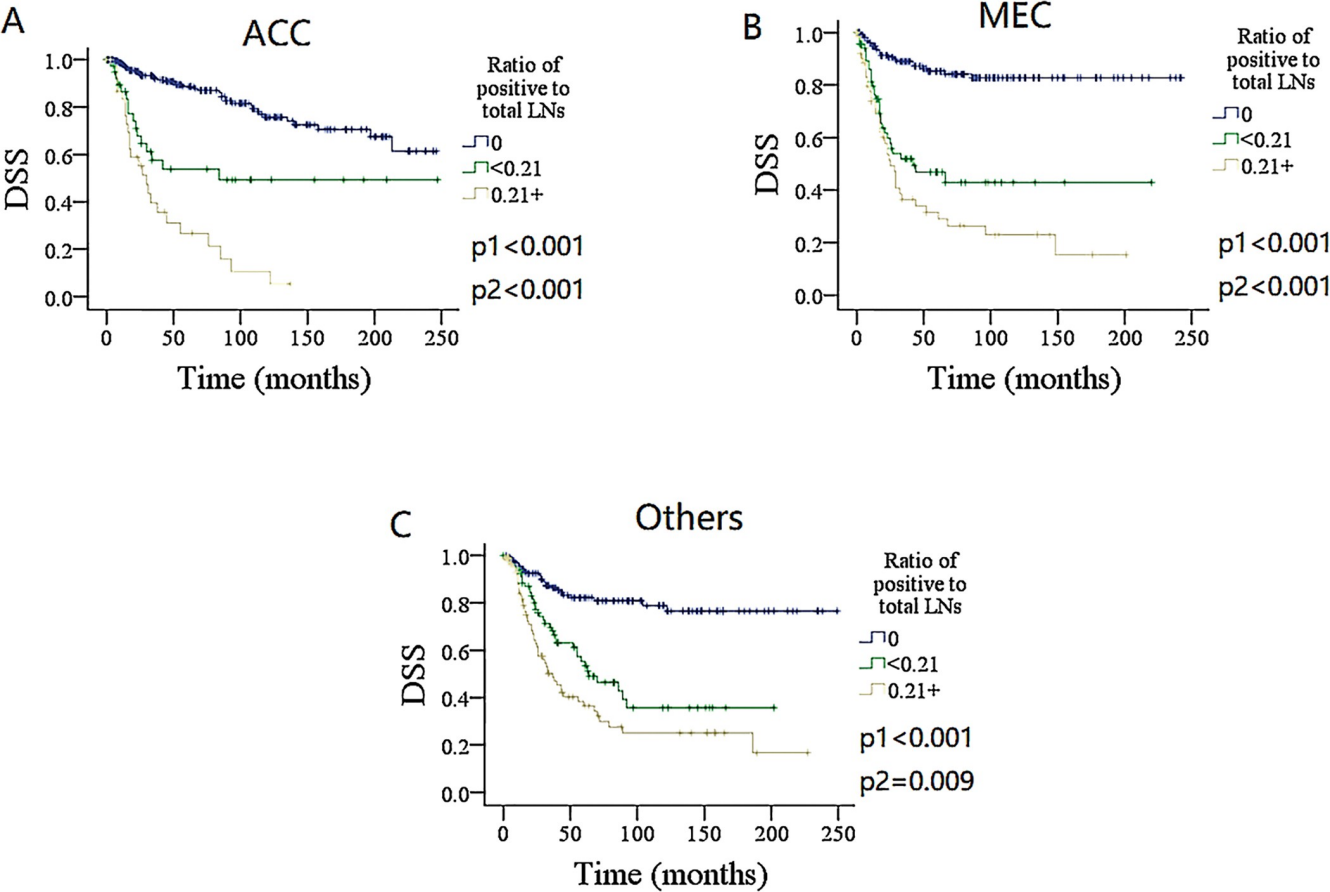
Comparison of disease specific survival (DSS) among patients with different ratio of metastatic lymph nodes (LNs) stratificated by pathologic types. A for adenoid cystic carcinoma (ACC), B for mucoepidermoid carcinoma (MEC), C for other types.

To furtherly validate the finding, the impact of the number and ratio of metastatic LNs on DSS and OS in different pathologic type groups was analyzed (Figs 4–7). Among the groups of ACC, MEC, and others, both the number and ratio of metastatic LNs were related to the prognosis, the group of 3+ positive LNs had the poorest survival, followed by groups of 1–2 metastatic LNs, and the presence of ratio ≥ 0.21 was associated with the most increased death risk.

## Discussion

The most interesting finding was that the number and ratio of metastatic LNs presented an important effect on DSS and OS in SGC independent of its pathologic type. Prognostic models based on the number (0 vs. 1–2 vs. 3+) and ratio (0 vs. < 0.21 vs. ≥ 0.21) of metastatic LNs carried better survival stratification than the AJCC N stage. Therefore, this study could provide additional aid in the clinical management of SGC.

The number of metastatic LNs attracted much attention in prognosis prediction in solid cancer. In a pan-cancer analysis [10], it was expected that with the LN metastasis burden increasing, mortality risk increased continuously, and the growth trend was most pronounced in the first five positive LNs and gradually flattened out afterward. A new N stage was constructed (0 vs. 1 vs. 2 vs. 3–4 vs. 5–7 vs. 8+) for the head and neck cancer, it showed good DSS stratification, but most of the samples were from non-SGC patients. Prior evidence had prompted researchers to evaluate the role of this variable in salivary gland cancer. In a study by Aro et al. [4], which included one of the largest sample sizes, the authors described there was a strong association between the number of metastatic LNs and survival, and when adjusted for the number of metastatic LNs, greater size, extranodal extension, and lower level involvement of LN it was no longer related to decreased survival. Qian et al. [5] found that the positive LN number > 4 predicted worse DSS and the impact remained independent despite accounting for other pathologic factors. A similar finding was also confirmed in other reports [6, 11–13]. However, all these studies were limited by a severe imbalance in the proportion of parotid cancer and SGC, together with the presence of intraparotid LN and biological difference, our study uncovered that metastatic LN burden was also an important prognostic factor in SGC, and the issue was rarely analyzed. Cho et al. [8] retrospectively enrolled 99 patients with SGC who underwent neck dissection, they noted that the negative effect of 4+ metastatic LNs on prognosis was the greatest among the three groups (0 vs. 1–3 vs. 4+) in univariate analysis, but the association disappeared in the multivariate analysis. A possible explanation was their small sample size of patients with 4+ positive LNs, which could not provide a significant result.

The ratio of metastatic LNs was another factor considering both the positive and total examined LNs. It was reflected by two aspects: the surgeon’s ability to clean up LNs and the pathologist’s ability to detect LNs [14]. Some researchers would like to comment that the ratio was superior to the number in prognosis prediction. Liu et al. [15] analyzed the results of 47 patients with ACC who underwent neck dissection and reported that ratio greater than 0.2 rather than number ≥ 4 was an independent prognostic factor for metastasis-free survival. A similar finding was also confirmed in high-grade salivary gland cancers [12]: the presence of 2+ metastatic LNs did not alter the DSS or OS compared with the 0/1 positive LN group, but a ratio > 0.04 was associated with an increased two-fold risk of death. Our study consistently validated the positivity of the ratio in survival prediction, but we might assume that the ratio and number could be complementary. If the detected LN number was relatively small, the ratio appeared to be better, whereas the number of metastatic LNs was superior in those with a relatively large number of total LNs [16].

The limitations of the AJCC N stage in predicting the prognosis of major salivary gland cancer were well recognized, and the main points of disagreement were the rare occurrence of contralateral LN metastasis and the conflicted significance of extranodal extension [3, 6, 15]. Several authors had introduced alternative N stages [3, 4], and although these new N classifications exhibited greater C-index than the AJCC N stage, they neither neglected the existence of ratio nor calculated the ratio in the Cox model. Our study might be the first to suggest that the Cox model based on the number and ratio of positive LNs has better hazard discrimination and consistency than the AJCC N stage. This finding carried essential practical benefits. First, the two variables were reproducible and objective with minimal ambiguity and could be easily obtained even in resource-limited medical centers; thus, the provider’s cost was not increased. Second, because the two variables were exact data but not a continuous number, major staging changes were minimized owing to arbitrary and meaningless differences in the LN factor. For example, the size of 3.0 cm in an LN was defined as N1 in the traditional N stage, and even an enlargement of 0.1 cm in the LN meant N2a, different treatment strategies were likely to be performed in the two groups. However, if patients with both N1 and N2a had only one positive LN and similar prognosis, an over-treatment was ultimately developed. Our proposed Cox model might eliminate this issue. Third, some researchers would question other LN attributes, including size and extranodal extension, which should also be incorporated into staging except for nodal count, but the magnitude of their impact on mortality was much smaller than quantitative nodal burden without exception. Therefore, it was believed that these factors would be more suited for being used as modifying secondary factors [10].

There were more than 20 pathologic types in salivary gland cancer, and each category tended to show low, intermediate, or high-grade features [17]. It was necessary to validate the prognostic significance of the LN factors in each type, and it remained unclear whether a novel N stage based on a specific type could be suitable for other types [18]. The important issues were not addressed properly. Only one report examined their N stage in a subgroup analysis of pathologic types [4]. We noted that the number and ratio of metastatic LNs still significantly influenced the prognosis independent of pathologic types, and the difference between DSS and OS was statistically significant between each of them. The ACC and MEC were the two most common pathologic types in salivary gland cancers, and it was reasonable to deduce that the number and ratio of metastatic LNs provide good survival stratification in all SGCs.

Limitations in current study must be acknowledged. First, this was a retrospective study, there was an inherent bias. Second, data regarding margin status and tumor extension was unavailable, which might have affected our survival analysis. Third, because our results were analyzed based on a United State population, it remains unclear whether the findings are suitable for other populations.

In conclusion, the number and ratio of metastatic LNs had an important effect on DSS and OS in SGC independent of pathologic type. Prognostic models based on the number (0 vs. 1–2 vs. 3+) and ratio (0 vs. < 0.21 vs. ≥ 0.21) of metastatic LNs carried better survival stratification than the AJCC N stage.

## Supporting information

S1 Data (ZIP)
